# Relationship between systolic blood pressure and all-cause mortality: a prospective study in a cohort of Chinese adults

**DOI:** 10.1186/s12889-017-4965-5

**Published:** 2018-01-05

**Authors:** Chunsheng Li, Youren Chen, Qiongbing Zheng, Weiqiang Wu, Zhichao Chen, Lu Song, Shasha An, Zhifang Li, Shuohua Chen, S. L. Wu

**Affiliations:** 1 Shantou University Medical College, Shantou, Guangdong China; 2 Department of Cardiology, Second Affiliated Hospital of Shantou University Medical College, Shantou, Guangdong China; 3 Graduate School, North China University of Science and Technology, Tangshan, China; 4 Department of Cardiology, Kailuan General Hospital, Tangshan, China

**Keywords:** Systolic blood pressure, All-cause mortality, J-shaped relationship, Prospective cohort study, Sex

## Abstract

**Background:**

The association between systolic blood pressure (SBP) and all-cause mortality in Chinese adults remains unclear. This study aimed to identify the relationship of SBP with all-cause mortality in Chinese men and women.

**Methods:**

One hundred twenty-one thousand eighty-two employees of the Kailuan Group Corporation, aged 18 or older, who participated in physical examination from 2006 to 2007 or from 2008 to 2009, were enrolled and followed up for all-cause mortality. The information used to ascertain the outcome of death during follow-up was extracted from provincial vital statistics offices, hospitalization records from the 11 hospitals, or medical records from medical insurance companies.

**Results:**

The average age was 50.06 ± 12.85 in the overall sample. Over 7 years of follow-up, 5945 participants, including 5520 men and 425 women had all-cause mortality. After multivariate adjustment, men in SBP group of <100, 120–139, 140–159, 160–179 and ≥180 mmHg had hazard ratios (HR) of 1.46 (1.14–1.86), 1.14 (1.04–1.26), 1.29 (1.16–1.44), 1.57 (1.38–1.79) and 2.07 (1.76–2.43), respectively, and displayed significantly increased risk of all-cause mortality compared to those with SBP in the range of 100–119 mmHg. Compared with the group of 100–119 mmHg, women in SBP group of 140–159, 160–179 and ≥180 mmHg had significantly greater risk with HRs of 1.44 (95% CI, 1.01–2.07), 1.63 (95% CI, 1.04–2.55) and 2.31 (95% CI, 1.27–4.20).

**Conclusions:**

Either lower (<100 mmHg) or higher (>120 mmHg) SBP was associated with an increased all-cause mortality risk and a J-shaped relationship was observed between SBP and all-cause mortality in men. Only SBP exceeding 140 mmHg was related to a higher risk in women. The relationship between SBP and all-cause mortality among Chinese adults may differ by sex.

**Electronic supplementary material:**

The online version of this article (10.1186/s12889-017-4965-5) contains supplementary material, which is available to authorized users.

## Background

High blood pressure is the most common non-communicable disease encountered in primary care that leads to complications such as cardiovascular diseases, chronic kidney disease and death [1]. The number of deaths due to cardiovascular diseases, probably caused by hypertension, accounted for almost a third of the global number of deaths in 2013 [2]. Moreover, a systematic analysis of the Global Burden of Disease Study reported that hypertension caused 37.9 million disability adjusted life years (DALYs) in Chinese population, including 2.3 million years lived with disability and 35.6 million years of life lost, which represented 12% of the overall DALYs in 2010 [3].

Up to date, the issue on the potential relationship of all-cause mortality with systolic blood pressure (SBP) remains controversial. The sixth report of the Joint National Committee on the Detection, Evaluation, and Treatment of Hypertension (JNC/6) recommended that optimum systolic blood pressure should be <120 mmHg [4]. The JNC/7 stated that the relationship between blood pressure and risk of cardiovascular events is continuous, consistent, and independent of other risk factors [5], which was based on the general belief that there is an inherent relationship between SBP and the risk of cardiovascular diseases [6]. Indeed, the concept that cardiovascular and all-cause mortality depend on SBP, in a monotonically increasing manner, was mainly derived from the utilization of linear logistic regression to estimate their relationship. Keys [7], however, argued that the relationship of total death and death due to coronary heart disease with SBP in men is non-linear, which was contrary to the result of Framingham’s study [6]. Furthermore, it was suggested that low SBP was associated with excess mortality, particularly for the elderly patients and patients with poor left ventricular function or with overtreatment [8–11]. Thus, a U-curve relationship was observed. The above conclusions were largely based on the population of Western countries, whereas data on the association between SBP and mortality among Asian population are limited.

A J-shaped association between lower SBP and all-cause mortality was previously shown in old and middle-aged Korean men [12]. Previously, the Shanghai Women’s Health Study indicated that SBP was positively correlated with all-cause mortality among Chinese women [13]. However, this cohort only included female participants. The relationship between SBP and mortality may differ by sex but its exact nature remains unclear among Chinese. Therefore, it is fundamental to seek for further evidence of the link between SBP and all-cause mortality among Chinese adults. To the best of our knowledge, no report on the J-shaped relationship between SBP and all-cause mortality in a large and relatively healthy population in China has been published so far. In addition, blood pressure of Chinese individuals may be different from that of individuals from other countries because of other behaviors and risk factors including smoking status, body mass index, physical activity, healthy dietary score, total cholesterol and fasting blood glucose [14]. Figuring out the definite threshold will be valuable for setting a SBP goal for Chinese hypertensive patients. Therefore, in this study we conducted a prospective analysis on a Chinese population in order to examine the relationship between SBP and all-cause mortality.

## Methods

### Study participants

In this study, a cohort of 126,847 working and retired employees of Kailuan Corporation in Tangshan was followed up for the occurrence of all-cause mortality. Tangshan is a large and littoral modern city located in the southeast of Beijing (China). The participants underwent medical examinations at the Kailuan General Hospital or its 10 affiliated hospitals in 2006 or in 2008. The employees were included in the study population if they met the following criteria: aged 18 years or older; having completed personal information and blood pressure data. Those with a history of stroke, myocardial infarction or cancer at baseline and without complete data related to the study were excluded. The average age was 50.06 ± 12.85 in the included participants. Written informed consent was obtained from each participant or its legal representative. The study was performed according to the guidelines of the Helsinki Declaration and was approved by the Ethics Committee of the Kailuan General Hospital.

### Data collection

Participants’ general data, including age, sex, education level, physical activity, alcohol intake, smoking status and history of hypertension, coronary heart disease, stroke, diabetes mellitus and use of antihypertensive drugs, were obtained via questionnaires administered by professional doctors at baseline. Height and body weight were also measured and body mass index (BMI) was calculated using the formula BMI = weight (kg)/ height (m^2^).

### Blood pressure measurement

Following the standard recommended procedures, SBP and diastolic blood pressure (DBP) were measured on the right upper arm of participants using a standardized mercury sphygmomanometer by a trained doctor after the participants avoided smoking or drinking coffee or tea for at least 30 min and had rested in sitting position for 15 min. The first and fifth Korotkoff sounds were recorded as SBP and DBP, respectively. Two readings each of SBP and DBP were taken at a 5-min interval. If these two readings differed by more than 5 mmHg, an additional measurement was performed. The average of these readings was considered as the examination BP.

### Biochemical measurements

Blood samples from participant’s antecubital vein were collected in EDTA tubes after an overnight fast. Plasma samples were separated by centrifugation at 3000 g for 10 min (centrifuge radius of 17 cm) at room temperature. The supernatant serum was decanted and used for measurements within 4 h. Measurement of fasting blood glucose (FBG) was performed using hexokinase/glucose-6-phosphate dehydrogenase method. Triglyceride (TG), high-density lipoprotein cholesterol (HDL-C) and low-density lipoprotein cholesterol (LDL-C) were enzymatically examined (inter-assay coefficient of variation <10%; Mind Bioengineering Co. Ltd., Shanghai, China). High-sensitivity (hs)-CRP was examined using high-sensitivity nephelometry assay (Cias Latex CRP-H, Kanto Chemical Co. Inc., Tokyo, Japan). An automatic biochemical analyzer (Hitachi 747; Hitachi, Tokyo, Japan) was used for measuring all biochemical variables at the central laboratory of the Kailuan General Hospital [14].

### Study outcomes

The follow-up began with the baseline examination from 2006 or 2008 through to December 31, 2014 or to the date of mortality or loss to follow-up. During follow-up we telephoned the participants to participate in the medical examinations in hospital. All participants were under continuous surveillance for incidence of all-cause mortality. Information used to ascertain the outcome of death during follow-up was extracted from provincial vital statistics offices, hospitalization records from the 11 hospitals, or medical records from medical insurance companies annually [15].

### Statistical analysis

Statistical analyses were conducted using SPSS 13.0 software (SPSS, Chicago, IL, USA) and the open source statistical software package R (version 3.20). Participants were classified into six groups based on SBP at baseline (group Q1 (SBP <100 mmHg); group Q2 (SBP 100–119 mmHg); group Q3 (SBP 120–139 mmHg); group Q4 (SBP 140–159 mmHg); group Q5 (SBP 160–179 mmHg) and group Q6 (SBP ≥180 mmHg)). Continuous variables were expressed as mean ± standard deviation (SD) and compared using one-way analysis of variance for unpaired samples of normally distributed parameters. Skewness distributed data (such as TG and hs-CRP) were converted by logarithmic transformation. Categorical variables were described by absolute numbers and percentages and the comparison between groups was conducted by chi-square test. The cumulative incidence of all-cause mortality was calculated using the “Life table” method and cumulative mortality rates were compared with a log-rank test. Hazard ratios (HR) and 95% confidence intervals (CI) of the incidence of all-cause mortality in relation to SBP levels were calculated for overall sample and sex stratified samples. Model 1 was an unadjusted model. Model 2 was adjusted for age, DBP, TG, LDL-C, HDL-C, FBG, BMI, hs-CRP, education level, physical activity, smoking status, alcohol consumption and use of antihypertensive drugs. In the overall sample, Model 2 was also used to control for the sex.

To further evaluate the associations between SBP and outcome in the sex stratified samples, HRs and 95% CIs were plotted. The plotted HRs were based on the covariate adjusted Cox proportional hazards model with a natural cubic splines transformation of blood pressure. We chose 116 and 162 mmHg as two knots basing on the maximum and minimum of SBP. Several sensitivity analyses were performed to examine whether the relationship between SBP and mortality was altered, excluding individuals with hypertension or diabetes mellitus history at baseline, those died within 1 year after baseline assessment or those with age ≥ 70, to examine whether the relationship between SBP and mortality was altered. Interactions between age, smoking status and SBP were tested respectively. Two-sided *p*-values <0.05 were accepted as statistically significant.

## Results

### Baseline characteristics

**Fig. 1:**
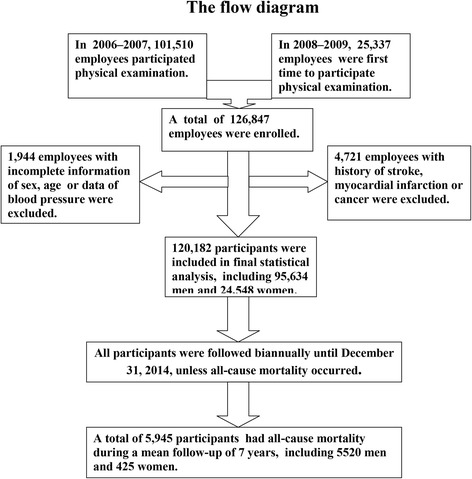
The flow diagram

Of 126,847 working and retired employees, 4721 with a prior history of stroke, myocardial infarction, or cancer and 1944 without available data related to the study at baseline were excluded. A total of 120,182 participants were finally enrolled in this prospective cohort study, including 95,634 men and 24,548 women (Fig. 1).

Differences in baseline participant characteristics across the six groups of SBP are presented in Table 1. Participants with higher SBP were generally older, more likely to be men, used antihypertensive drugs and had higher levels of DBP, TG, LDL-C, HDL-C, FBG, BMI and hs-CRP. Statistically significant differences among the SBP groups were also found for the following variables: education, physical activity, smoking status and alcohol consumption (*P* **<** 0.01). These associations confirmed the need for adjusting variables that confound the associations of SBP with all-cause mortality.

**Table 1:** Participants baseline characteristics according to systolic blood pressure groups

	Systolic pressure groups						P
	Q1	Q2	Q3	Q4	Q5	Q6	
	<100 mmHg	100–119 mmHg	120–139 mmHg	140–159 mmHg	160–179 mmHg	≥180 mmHg	
Participants, n	3562	29,723	48,420	25,273	9918	3286	
Men, n (%)	1801 (50.6)	20,740 (69.8)	40,066 (82.7)	21,652 (85.7)	8481 (85.5)	2894 (88.1)	<0.0001
Age, years	41.8 ± 12.7	44.9 ± 12.4	49.4 ± 12.34	54.7 ± 11.7	57.2 ± 11.0	58.7 ± 10.5	<0.0001
SBP, mm Hg	92.4 ± 4.5	109.5 ± 5.6	126.3 ± 5.8	145.7 ± 5.8	164.5 ± 5.6	189.4 ± 12.5	<0.0001
DBP, mm Hg	62.8 ± 5.2	73.9 ± 6.4	83.4 ± 6.8	90.9 ± 8.8	97.8 ± 10.5	106.9 ± 14.1	<0.0001
(lg)TG, mmol/L	−0.01 ± 0.24	0.06 ± 0.26	0.14 ± 0.27	0.18 ± 0.27	0.19 ± 0.26	0.21 ± 0.27	<0.0001
LDL-C, mmol/L	2.2 ± 0.8	2.4 ± 0.9	2.4 ± 1.0	2.5 ± 1.0	2.5 ± 1.2	2.5 ± 1.0	<0.0001
HDL-C, mmol/L	1.5 ± 0.4	1.5 ± 0.4	1.5 ± 0.4	1.6 ± 0.4	1.6 ± 0.6	1.6 ± 0.4	<0.0001
FBG, mmol/L	5.0 ± 1.3	5.2 ± 1.4	5.5 ± 1.6	5.7 ± 1.8	5.9 ± 2.0	5.9 ± 2.0	<0.0001
BMI, kg/m^2^	22.5 ± 3.1	23.8 ± 3.2	25.0 ± 3.4	25.9 ± 3.5	26.2 ± 3.5	26.4 ± 3.8	<0.0001
(lg)hs-CRP, mmol/L	−0.18 ± 0.62	−0.13 ± 0.64	−0.08 ± 0.65	0.02 ± 0.64	0.06 ± 0.63	0.10 ± 0.64	<0.0001
Education, n (%)							<0.0001
Illiteracy/primary	164 (4.9)	1845 (6.5)	4061 (8.7)	3163 (13.0)	1498 (15.6)	592 (18.4)	
Middle school	2443 (72.5)	22,625 (79.2)	39,257 (83.9)	20,165 (82.9)	7887 (82.0)	2566 (79.6)	
College/University	764 (22.7)	4079 (14.3)	3489 (7.5)	995 (4.1)	230 (2.4)	66 (2.0)	
Physical activity 3 times/week, n (%)	352 (10.6)	3589 (12.8)	6753 (14.7)	4277 (18.0)	1845 (19.7)	648 (20.6)	<0.0001
Past/current alcohol drinker, n(%)	1229 (36.4)	12,122 (42.3)	20,320 (43.2)	9997 (40.7)	3582 (37.1)	1256 (38.8)	<0.0001
Past/current smoker, n(%)	1086 (32.1)	11,582 (40.4)	19,771 (42.1)	9868 (40.2)	3587 (37.1)	1274 (39.4)	<0.0001
Use of antihypertensives, n(%)	17 (0.5)	366 (1.2)	1750 (3.6)	2629 (10.4)	1796 (18.1)	768 (23.4)	<0.0001
Diuretic, n(%)	3 (0.1)	82 (0.3)	419 (0.9)	541 (2.1)	339 (3.4)	129 (3.9)	<0.0001
CCB, n(%)	5 (0.1)	84 (0.3)	373 (0.8)	591 (2.3)	358 (3.6)	146 (4.4)	<0.0001
ACEI/ARB, n(%)	4 (0.1)	86 (0.3)	336 (0.7)	544 (2.2)	313 (3.2)	137 (9.6)	<0.0001
β-Blockers, n(%)	0 (0)	40 (0.1)	143 (0.3)	202 (0.8)	110 (1.1)	59 (1.8)	<0.0001

*Abbreviations*: *SBP* systolic blood pressure, *DBP* diastolic blood pressure, *TG* triglyceride, *LDL-C* low-density lipoprotein cholesterol, *HDL-C* high-density lipoprotein cholesterol, *FBG* fasting blood glucose, *BMI* body mass index, *hs-CRP* high sensitivity C-reactive protein. TG and hs-CRP were converted by logarithmic transformation

### Incidence of all-cause mortality in different groups of systolic pressure

**Fig. 2:**
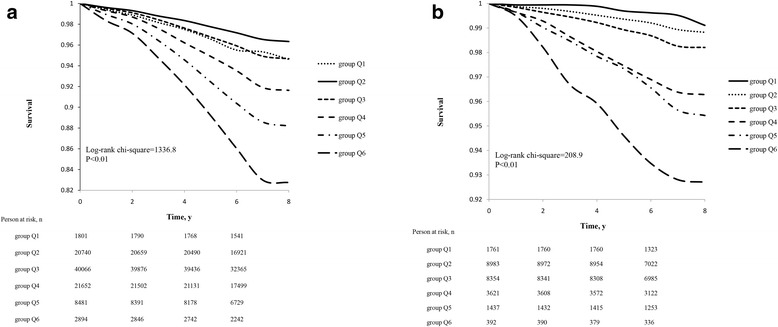
Survival plots of all-cause mortality in relation to the different groups of systolic blood pressure in (**a**) the men and (**b**) the women

Overall, 5945 participants had all-cause death during an average follow-up of 7 years, including 5520 men and 425 women. There were 90, 731, 1972, 1719, 954 and 479 deaths among the six SBP groups, respectively, in overall sample, with cumulative mortality rates of 3.3%, 2.9%, 4.7%, 7.7%, 10.7% and 16.0%. The sex specific analysis revealed that men with SBP of 100**–**119 mmHg had the lowest cumulative mortality rate (3.7%) and women with SBP < 100 mmHg had the lowest cumulative mortality rate (0.9%) (Table 2). Significant differences in the cumulative incidence rates of all-cause mortality were observed among the SBP groups in the overall sample and in sex stratified samples (χ^2^ = 1772.8, *P* < 0.01 for the overall sample, χ^2^ = 1336.8, P < 0.01 for men and χ^2^ = 208.9, P < 0.01 for women). The survival plots, with the actual cases of survival in men and women, were respectively presented in Fig. 2a and b.

**Table 2:** Hazard ratios (HR) and 95% confidence intervals (95% CI) of all-cause mortality according to systolic blood pressure groups

	Systolic pressure groups						P for trend
	Q1	Q2	Q3	Q4	Q5	Q6	
	<100 mmHg	100–119 mmHg	120–139 mmHg	140–159 mmHg	160–179 mmHg	≥180 mmHg	
General population							
Cumulative	90 (3.3)	731 (2.9)	1972 (4.7)	1719 (7.7)	954 (10.7)	479 (16.0)	
Mortality, n(%)							
Model 1	1.03 (0.83–1.29)	1	1.65 (1.52–1.80)	2.76 (2.53–3.01)	3.95 (3.59–4.35)	6.12 (5.45–6.86)	<0.0001
Model 2	1.30 (1.03–1.64)	1	1.13 (1.03–1.24)	1.29 (1.17–1.44)	1.57 (1.39–1.78)	2.09 (1.79–2.44)	<0.0001
Gender subgroup							
Male							
Cumulative	83 (5.4)	651 (3.7)	1843 (5.3)	1596 (8.4)	895 (11.8)	452 (17.3)	
Mortality, n(%)							
Model 1	1.45 (1.15–1.82)	1	1.47 (1.35–1.61)	2.37 (2.16–2.60)	3.44 (3.11–3.81)	5.20 (4.61–5.87)	<0.0001
Model 2#	1.46 (1.14–1.86)	1	1.14 (1.04–1.26)	1.29 (1.16–1.44)	1.57 (1.38–1.79)	2.07 (1.76–2.43)	<0.0001
Female							
Cumulative	7 (0.9)	80 (1.1)	129 (1.8)	123 (3.6)	59 (4.6)	27 (7.2)	
Mortality, n(%)							
Model 1	0.46 (0.21–0.99)	1	1.68 (1.27–2.22)	3.64 (2.75–4.83)	4.40 (3.14–6.15)	7.48 (4.84–11.58)	<0.0001
Model 2#	0.56 (0.24–1.29)	1	1.02 (0.75–1.39)	1.44 (1.01–2.07)	1.63 (1.04–2.55)	2.31 (1.27–4.20)	0.017

Model 1: unadjusted

Model 2: adjusted for age, gender, diastolic blood pressure (DBP), triglycerides (TG), low-density lipoprotein cholesterol (LDL-C), high-density lipoprotein cholesterol (HDL-C), fasting blood glucose (FBG), body mass index (BMI), high-sensitivity C-reactive protein (hs-CRP), education level, physical activity, smoking status, alcohol consumption and use of antihypertensives

Model 2#: adjusted for age, DBP, TG, LDL-C, HDL-C, FBG, BMI, hs-CRP, education level, physical activity, smoking status, alcohol consumption and use of antihypertensives

### Hazard ratios for all-cause mortality in different groups of systolic pressure

In the unadjusted Cox proportional hazards analysis, groups of SBP ≥ 120 mmHg were all significantly associated with a greater risk of all-cause mortality compared with the reference group with SBP of 100**–**119 mmHg in the overall sample (all *P* values <0.05). Adjustment for the confounding factors confirmed the excess mortality risk in the groups of SBP ≥ 120 mmHg with 1.13- to 2.02-fold HRs (all *P* values <0.05) in Model 2. Interestingly, participants with SBP < 100 mmHg appeared to be at significantly higher risk of death (HR = 1.30, 95% CI 1.03–1.64) in Model 2. Moreover, participants with both lower (<100 mmHg) and higher (≥120 mmHg) SBP had a statistically significant trend toward an increased risk of mortality (P for trend <0.01) (Table 2).

After controlling for all confounders, Model 2 indicated that compared with the reference group with SBP of 100**–**119 mmHg, men in the groups with SBP of <100, 120–139, 140–159, 160–179 and ≥180 mmHg continued to have a significantly higher mortality with HRs of 1.46 (95% CI, 1.14–1.86), 1.14 (95% CI, 1.04–1.26), 1.29 (95% CI, 1.16–1.44), 1.57 (95% CI, 1.38–1.79), 2.07 (95% CI, 1.76–2.43) (all *P* values <0.05, P for trend <0.01), respectively. However, among women, only the participants in the SBP groups of 140–159, 160–179 and ≥180 mmHg, compared with those in the group of 100–119 mmHg in Model 2, had a significantly increased risk for death with HRs of 1.44 (95% CI, 1.01–2.07), 1.63 (95% CI, 1.04–2.55), 2.31 (95% CI, 1.27–4.20) (all P values <0.05, P for trend = 0.017), respectively (Table 2).

**Fig. 3:**
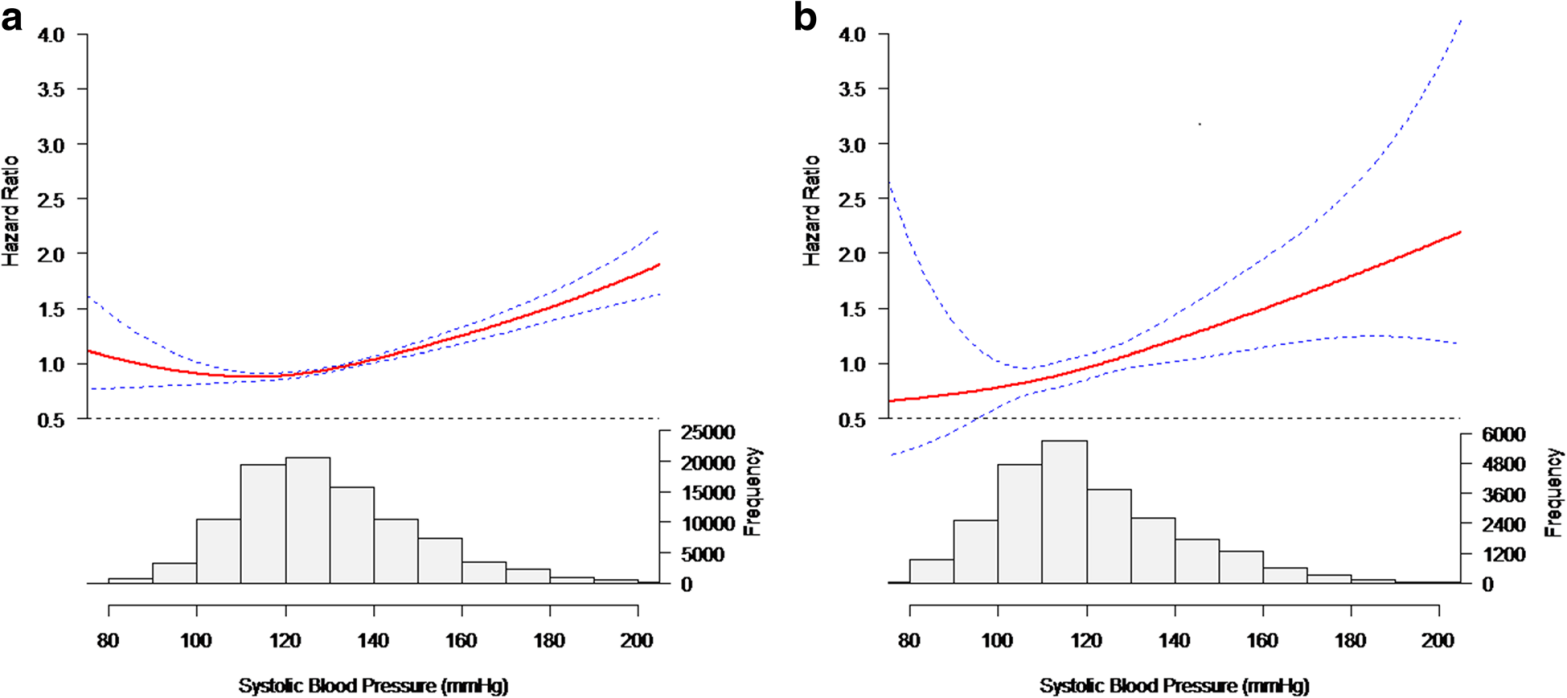
Systolic blood pressure and all-cause mortality in (**a**) the men and (**b**) the women. Results were obtained by multivariable Cox regression with natural cubic splines. Solid line represents the estimated hazard ratio of all-cause mortality with upper and lower 95% confidence intervals denoted by broken lines. The histogram below each spline indicates the major distribution of systolic blood pressure

We further conducted the natural cubic spline analysis with adjustment for covariates to confirm the J-shaped relationship between SBP and the outcome. The results revealed a J-shaped relationship between SBP and all-cause mortality risk with increased hazard rates at low and high SBPs in the men (Fig. 3a). The nadir SBP, where the risk of death was the lowest, was 110 mmHg in the men. However, there was no hint of a J-curve association of death with SBP in the women (Fig. 3b). Notably, sex-based differences were observed.

### Sensitivity analyses

After omission of 12,957 participants with pre-existing hypertension (Additional file 1: Table S1) or 3348 with pre-existing diabetes mellitus at baseline (Additional file 2: Table S2) or 238 who died within 1 year after baseline (Additional file 3: Table S3) or 9102 who were aged 70 or older (Additional file 4: Table S4**)**, similar results were found in sex stratified samples after we had repeated all models. The J-shaped relationship between SBP and all-cause mortality was recorded in the men, whereas this relationship was not found in the women. Interactions between age, smoking status and SBP were also tested (both *P*-values <0.001). Similar findings were shown in male adults aged 60 or older and male non-smokers (Additional file 5: Table S5).

## Discussion

Our analysis indicated that the lowest all-cause mortality rate was in the SBP range of 100–119 mmHg in the men. Controlling for covariates, the risk tended to significantly increase with SBP either <100 mmHg or **≥**120 mmHg. Because of the imbalance in sex distribution with more men than women in this cohort, a similar result was showed in the overall sample.

In the Framingham study, it was found that cardiovascular and total mortality depend on SBP in a continuously increasing manner [6]. The SPRINT trial has recently demonstrated that in hypertensive adults without diabetes, a SBP goal of <120 mmHg resulted in 25% and 27% reduction in cardiovascular events and mortality, respectively, compared with a SBP goal of <140 mmHg [16]. Both these studies partially supported our data. The ACCORD trial, however, previously suggested that no significant reduction in cardiovascular events in patients with type 2 diabetes was achieved by tight lowering of SBP (<120 mmHg), compared with the standard group in which SBP remained above 130 mmHg. Differently, only patients with diabetes were enrolled in the ACCORD trial, whereas patients with diabetes were excluded in the SPRINT.

However, the SPRINT has yet to determine the relationship between lower SBP (<100 mmHg) and overall death. Conversely, our data implied that SBP below 100 mmHg was related to the outcome with a 46% higher risk in men, providing evidence to address the controversial issue on the potential harms including all-cause mortality with lowering SBP beyond a certain threshold. Although the excess mortality rate was previously reported at low SBP levels by several studies, participants in these studies were majorly elderly individuals and had cardiovascular diseases [10, 11, 17–21]. To the best of our knowledge there was insufficient evidence from a relatively healthy population of Chinese adults.

Our sex-specific analysis also demonstrated a continuous increase in mortality rates across the SBP groups for women. With adjustment for covariates, a significantly increased risk of death with SBP of ≥140 mmHg without any indication of greater risk among groups of SBP < 140 mmHg was recorded. Similar results were found by Port et al. [22]. In our study, it should be noted that SBP values of 120–139 mmHg and below 100 mmHg were both associated with increased risk in men rather than women. Indeed, there was a sex-dependent background risk of mortality that is independent of SBP [22]. Current evidence-based guidelines [1, 23] for the management of high blood pressure in adults mainly focus on age, ethnicity and co-morbidities but ignore sex-related effects. Clearly, there was more evidence for tight control of SBP (100–119 mmHg) in men than women in our models, whereas prospective trials to address safety and efficacy of different SBP goals for each sex with or without hypertension are essentially needed.

In our study, participants with higher SBP tended to be older, were more commonly male, generally used antihypertensive drugs and had higher levels of DBP, TG, LDL-C, HDL-C, FBG, BMI and hs-CRP. Other covariates, including education, physical activity, smoking status and alcohol consumption, were also found to be significantly different among the SBP groups. Thus, we need to adjust these covariates in Cox proportional hazards model to reduce bias in estimating the effect of SBP on the outcome. After adjustment for these covariates, we found that a J-shaped curve relationship with elevated risk both at lower (<100 mmHg) and higher (≥120 mmHg) SBPs exists between SBP and all-cause mortality in men even though those aged 70 or older were excluded, while a positive association of SBP with outcome was seen in women. Since the majority of participants were male, the incidence rates of mortality were similar in the general and male population. Moreover, age-specific impact on death independent of SBP might not be neglected. Our sensitivity analysis suggested that the J-curve relationship between SBP and death remained in male population aged 60 or older. Concordant with our results, Keys [7] argued that the relationship between SBP and overall death is non-linear in men. Two Korean studies have shown similar evidence on the J-shaped association between lower SBP (<100 mmHg) and all-cause mortality based on an aged population [12, 24]. Previous investigation in America indicated that all-cause mortality was higher in the low (less than 130 mmHg) than the middle (130–159 mmHg) SBP group for participants aged 65–79 years [20]. Another study showed that SBP bears a U-shaped relationship with the lowest mortality rate in the range of 120–139 mmHg among patients with heart failure [25]. The sensitivity analysis in the present study showed that the effects of SBP on mortality in relatively healthy population vary by sex, age and even smoking status, thus these factors should be taken into consideration when defining a systolic goal.

One of the most widely cited theories on the J-curve epiphenomenon is that it may reflect a severe and/or debilitating underlying chronic condition including hypertension and diabetes mellitus thereby increasing mortality [26, 27]. However, in the present study the sensitivity analyses revealed a similar result after exclusion of participants who were with pre-existing hypertension or diabetes mellitus at entry. This result suggested that lower SBP also increases the risk of death in relatively healthy male population. In addition, lower SBP may be a sign of heart failure or poor contractile reserve in the environment of decompensated or advanced left ventricular systolic dysfunction since SBP is dependent on cardiac output [11]. Flack et al. argued that a J-shaped relationship between SBP and mortality exists in elderly individuals during short but not during prolonged follow-up, attributable to low SBP near the time of death [28]. The sensitivity analyses in our study showed that exclusion of the participants with <1 year of follow-up did not alter the shape of the J-curve. Thus, low SBP may affect long-term outcome in relatively healthy population.

Our study has some strengths, including the prospective design, standardized criteria and protocols for assessing blood pressure which should minimize potential bias. We included a detailed follow-up with confirmation of mortality through reviewing medical records. More importantly, the sex-specific analysis showed a J-shaped relationship of SBP with all-cause mortality among Chinese males in the absence of any previous literature on this issue. However, our study also had limitations. Firstly, the sample was based on a single community rather than a nationally representative population, so our results may not be generalizable to all adults in China. Secondly, although we adjusted our analysis for baseline confounders, any unmeasured confounders could have been missed. Thirdly, we assigned the same SBP value in statistical analysis, which may have simplified and underestimated the actual association between SBP and the outcome. Finally, though it has been suggested that sex differences in various cardiovascular diseases could be influenced by sex differences [29], the mechanism underlying the sex-specific association between all-cause mortality and SBP cannot be explained in our findings.

## Conclusion

The relationship between SBP and all-cause mortality in the men followed a J-shaped curve, with significant higher risk of all-cause mortality in the low (<100 mmHg) and high (>120 mmHg) SBP levels. Only SBP exceeding 140 mmHg was associated with increased mortality risk in the women. The relationship of SBP with all-cause mortality among Chinese adults may differ by sex. The present findings will be contributive in monitoring hypertensive patients.

## Additional files

Additional file 1: Supplementary Table S1. Hazard ratios (HR) and 95% confidence intervals (95% CI) of all-cause mortality according to systolic blood pressure groups among participants with no history of hypertension. (DOC 52 kb)

Additional file 2: Supplementary Table S2. Hazard ratios (HR) and 95% confidence intervals (95% CI) of all-cause mortality according to systolic blood pressure groups among participants with no history of diabetes mellitus. (DOC 53 kb)

Additional file 3: Supplementary Table S3. Hazard ratios (HR) and 95% confidence intervals (95% CI) of all-cause mortality according to systolic blood pressure groups from 1 year after baseline. (DOC 52 kb)

Additional file 4: Supplementary Table S4. Hazard ratios (HR) and 95% confidence intervals (95% CI) of all-cause mortality according to systolic blood pressure groups after exclusion of individuals who were older than 70 years. (DOC 52 kb)

Additional file 5: Supplementary Table S5. Hazard ratios (HR) and 95% confidence intervals (95% CI) of all-cause mortality according to systolic blood pressure groups stratified by age and smoking status. (DOC 67 kb)

## Abbreviations

BMI: Body mass index
BP: Blood pressure
CI: Confidence interval
DALY: Disability adjusted life years
DBP: Diastolic blood pressure
FBG: Fasting blood glucose
HDL-C: High-density lipoprotein cholesterol
HR: Hazard ratio
hs-CRP: High sensitivity C-reactive protein
JNC/6: The Sixth Joint National Committee
JNC/7: The Seventh Joint National Committee
LDL-C: Low-density lipoprotein cholesterol
SBP: Systolic blood pressure
TG: Triglyceride

## References

[1] James PA, Oparil S, Carter BL, Cushman WC, Dennison-Himmelfarb C, Handler J, Lackland DT, LeFevre ML, MacKenzie TD, Ogedegbe O (2014). 2014 evidence-based guideline for the management of high blood pressure in adults: report from the panel members appointed to the eighth joint National Committee (JNC 8). JAMA.

[2] Collaborators GMaCoD (2015). Global, regional, and national age–sex specific all-cause and cause-specific mortality for 240 causes of death, 1990–2013: a systematic analysis for the global burden of disease study 2013. Lancet.

[3] Lozano R, Naghavi M, Foreman K, Lim S, Shibuya K, Aboyans V, Abraham J, Adair T, Aggarwal R, Ahn SY (2012). Global and regional mortality from 235 causes of death for 20 age groups in 1990 and 2010: a systematic analysis for the global burden of disease study 2010. Lancet.

[4] The sixth report of the joint National Committee on prevention, detection, evaluation, and treatment of high blood pressure. Arch Intern Med. 1997;157(21):2413–46. 10.1001/archinte.157.21.2413 9385294

[5] Chobanian AV, Bakris GL, Black HR, Cushman WC, Green LA, Izzo JL, Jones DW, Materson BJ, Oparil S, Wright JT (2003). The seventh report of the joint National Committee on prevention, detection, evaluation, and treatment of high blood pressure: the JNC 7 report. JAMA.

[6] Stokes J, Kannel WB, Wolf PA, D'Agostino RB, Cupples LA. Blood pressure as a risk factor for cardiovascular disease. The Framingham Study--30 years of follow-up. Hypertension. 1989;13(5 Suppl):I13–8. 10.1161/01.hyp.13.5_suppl.i13 2535213

[7] AB K: Seven countries: a multivariate analysis of death and coronary heart disease**.** Cambridge, MA: Harvard University Press 1980, 381.

[8] Group JS (2008). Principal results of the Japanese trial to assess optimal systolic blood pressure in elderly hypertensive patients (JATOS). Hypertension Res: Official J Japanese Society Hypertension.

[9] Ogihara T, Saruta T, Rakugi H, Matsuoka H, Shimamoto K, Shimada K, Imai Y, Kikuchi K, Ito S, Eto T (2010). Target blood pressure for treatment of isolated systolic hypertension in the elderly: valsartan in elderly isolated systolic hypertension study. Hypertension.

[10] Kannel WB, D'Agostino RB, Silbershatz H (1997). Blood pressure and cardiovascular morbidity and mortality rates in the elderly. Am Heart J.

[11] Ather S, Chan W, Chillar A, Aguilar D, Pritchett AM, Ramasubbu K, Wehrens XH, Deswal A, Bozkurt B (2011). Association of systolic blood pressure with mortality in patients with heart failure with reduced ejection fraction: a complex relationship. Am Heart J.

[12] Yi SW, Ohrr H (2015). Low systolic blood pressure and mortality from all causes and vascular diseases among older middle-aged men: Korean veterans health study. J Preventive Med Public Health = Yebang Uihakhoe chi.

[13] Dorjgochoo T, Shu XO, Zhang X, Li H, Yang G, Gao L, Cai H, Gao YT, Zheng W (2009). Relation of blood pressure components and categories and all-cause, stroke and coronary heart disease mortality in urban Chinese women: a population-based prospective study. J Hypertens.

[14] Liu Y, Chi H-j, Cui L-f, Yang X-c, Wu Y-t, Huang Z, Zhao H-y, Gao J-s, Wu S-l, Cai J (2014). The ideal cardiovascular health metrics associated inversely with mortality from all causes and from cardiovascular diseases among adults in a northern Chinese industrial city. PLoS One.

[15] Zhang Q, Zhou Y, Gao X, Wang C, Zhang S, Wang A, Li N, Bian L, Wu J, Jia Q (2013). Ideal cardiovascular health metrics and the risks of ischemic and intracerebral hemorrhagic stroke. Stroke.

[16] Group SR (2015). A randomized trial of intensive versus standard blood-pressure control. N Engl J Med.

[17] Lindholm L, Lanke J, Bengtsson B (1986). U-shaped association between mortality and blood pressure in a thirteen-year prospective study. Fam Pract.

[18] Siegel D, Kuller L, Lazarus NB, Black D, Feigal D, Hughes G, Schoenberger JA, Hulley SB (1987). Predictors of cardiovascular events and mortality in the systolic hypertension in the elderly program pilot project. Am J Epidemiol.

[19] Coope J, Warrender TS, McPherson K (1988). The prognostic significance of blood pressure in the elderly. J Hum Hypertens.

[20] Taylor JO, Cornoni-Huntley J, Curb JD, Manton KG, Ostfeld AM, Scherr P, Wallace RB (1991). Blood pressure and mortality risk in the elderly. Am J Epidemiol.

[21] Rosman Y, Kopel E, Shlomai G, Goldenberg I, Grossman E (2015). The association between admission systolic blood pressure of heart failure patients with preserved systolic function and mortality outcomes. European journal of internal medicine.

[22] Port S, Demer L, Jennrich R, Walter D, Garfinkel A (2000). Systolic blood pressure and mortality. Lancet.

[23] Mancia G, Fagard R, Narkiewicz K, Redon J, Zanchetti A, Bohm M, Christiaens T, Cifkova R, De Backer G, Dominiczak A (2013). 2013 ESH/ESC guidelines for the management of arterial hypertension: the task force for the management of arterial hypertension of the European Society of Hypertension (ESH) and of the European Society of Cardiology (ESC). J Hypertens.

[24] Yi SW, Hong S, Ohrr H (2015). Low systolic blood pressure and mortality from all-cause and vascular diseases among the rural elderly in Korea; Kangwha cohort study. Medicine.

[25] Lee DS, Ghosh N, Floras JS, Newton GE, Austin PC, Wang X, Liu PP, Stukel TA, Tu JV (2009). Association of blood pressure at hospital discharge with mortality in patients diagnosed with heart failure. Circulation Heart Failure.

[26] Glynn RJ, Field TS, Rosner B, Hebert PR, Taylor JO, Hennekens CH (1995). Evidence for a positive linear relation between blood pressure and mortality in elderly people. Lancet.

[27] Boshuizen HC, Izaks GJ, van Buuren S, Ligthart GJ (1998). Blood pressure and mortality in elderly people aged 85 and older: community based study. BMJ.

[28] Flack JM, Neaton J, Grimm R, Shih J, Cutler J, Ensrud K, MacMahon S (1995). Blood pressure and mortality among men with prior myocardial infarction: multiple risk factor intervention trial research group. Circulation.

[29] Gohar EY, Giachini FR, Pollock DM, Tostes RC (2016). Role of the endothelin system in sexual dimorphism in cardiovascular and renal diseases. Life Sci.

