# Supplementary material for: Relationship between systolic blood pressure and all-cause mortality: a prospective study in a cohort of Chinese adults
Source: BMC Public Health. 2018 Jan 5;18:107. doi: 10.1186/s12889-017-4965-5 (PMC5756411; doi:10.1186/s12889-017-4965-5)
Supplement: Supplementary file 1 — Supplementary Table S1. Hazard ratios (HR) and 95% confidence intervals (95% CI) of all-cause mortality according to systolic blood pressure groups among participants with no history of hypertension. (DOC 52 kb) [file 12889_2017_4965_MOESM1_ESM.doc]

| **Supplementary Table S1 Hazard ratios (HR) and 95% confidence intervals (95% CI) of all-cause mortality according to systolic blood pressure groups among participants with no history of hypertension** | | | | | | | |
| --- | --- | --- | --- | --- | --- | --- | --- |
|  | **Systolic pressure groups** | | | | | | **P for trend** |
|  | **Q1** | **Q2** | **Q3** | **Q4** | **Q5** | **Q6** |  |
|  | **<100mm Hg** | **100–119mm Hg** | **120–139mm Hg** | **140–159mm Hg** | **160–179mm Hg** | **≥180mm Hg** |  |
| **Overall sample** |  |  |  |  |  |  |  |
| **cumulative mortality ,n(%)** | **87 (3.2)** | **685 (2.8)** | **1773 (4.6)** | **1329 (7.3)** | **632 (10.2)** | **275 (15.1)** |  |
| **Model 1** | **1.05 (0.84–1.32)** | **1** | **1.66 (1.52–1.81)** | **2.74 (2.50–3.01)** | **3.87 (3.48–4.32)** | **6.06 (5.27–6.97)** | **<0.0001** |
| **Model 2** | **1.30 (1.02–1.64)** | **1** | **1.16 (1.06–1.28)** | **1.34 (1.19–1.50)** | **1.62 (1.40–1.86)** | **2.13 (1.77–2.56)** | **<0.0001** |
| **Sex stratified sample** |  |  |  |  |  |  |  |
| **Male** |  |  |  |  |  |  |  |
| **cumulative mortality ,n(%)** | **80 (5.3)** | **612 (3.5)** | **1674 (5.2)** | **1236 (7.9)** | **604 (11.1)** | **262 (15.9)** |  |
| **Model 1** | **1.47 (1.16–1.85)** | **1** | **1.49 (1.36–1.63)** | **2.31 (2.09–2.54)** | **3.33 (2.97–3.72)** | **5.03 (4.35–5.81)** | **<0.0001** |
| **Model 2#** | **1.44 (1.13–1.84)** | **1** | **1.18 (1.07–1.30)** | **1.32 (1.17–1.48)** | **1.62 (1.40–1.87)** | **2.09 (1.73–2.54)** | **<0.0001** |
| **Female** |  |  |  |  |  |  |  |
| **cumulative mortality,n(%)** | **7 (0.9)** | **73 (1.0)** | **99 (1.5)** | **93 (3.8)** | **28 (3.8)** | **13 (7.5)** |  |
| **Model 1** | **0.49 (0.23–1.07)** | **1** | **1.50 (1.11–2.04)** | **4.06 (2.99–5.52)** | **3.80 (2.46–5.87)** | **8.11 (4.50–14.63)** | **<0.0001** |
| **Model 2#** | **0.56 (0.24–1.31)** | **1** | **0.97 (0.69–1.37)** | **1.77 (1.18–2.66)** | **1.68 (0.94–2.97)** | **2.93 (1.34–6.43)** | **0.002** |
| Model 1: unadjusted.  Model 2: adjusted for age, gender, diastolic blood pressure (DBP), triglycerides (TG), low-density lipoprotein cholesterol (LDL-C), high-density lipoprotein cholesterol (HDL-C), fasting blood glucose (FBG), body mass index (BMI), high-sensitivity C-reactive protein (hs-CRP), education level, physical activity, smoking status, alcohol consumption and use of antihypertensives.  Model 2#: adjusted for age, DBP, TG, LDL-C, HDL-C, FBG, BMI, hs-CRP, education level, physical activity, smoking status, alcohol consumption and use of antihypertensives. | | | | | | | |
