# Supplementary material for: Relationship between systolic blood pressure and all-cause mortality: a prospective study in a cohort of Chinese adults
Source: BMC Public Health. 2018 Jan 5;18:107. doi: 10.1186/s12889-017-4965-5 (PMC5756411; doi:10.1186/s12889-017-4965-5)
Supplement: Supplementary file 2 — Supplementary Table S2. Hazard ratios (HR) and 95% confidence intervals (95% CI) of all-cause mortality according to systolic blood pressure groups among participants with no history of diabetes mellitus. (DOC 53 kb) [file 12889_2017_4965_MOESM2_ESM.doc]

| **Supplementary Table S2 Hazard ratios (HR) and 95% confidence intervals (95% CI) of all-cause mortality according to systolic blood pressure groups among participants with no history of diabetes mellitus** | | | | | | | |
| --- | --- | --- | --- | --- | --- | --- | --- |
|  | **Systolic pressure groups** | | | | | | **P for trend** |
|  | **Q1** | **Q2** | **Q3** | **Q4** | **Q5** | **Q6** |  |
|  | **<100mm Hg** | **100–119mm Hg** | **120–139mm Hg** | **140–159mm Hg** | **160–179mm Hg** | **≥180mm Hg** |  |
| **Overall sample** |  |  |  |  |  |  |  |
| **cumulative mortality ,n(%)** | **84 (3.1)** | **676 (2.7)** | **1857 (4.6)** | **1607 (7.4)** | **879 (10.4)** | **444 (15.6)** |  |
| **Model 1** | **1.04 (0.82–1.30)** | **1** | **1.69 (1.55–1.85)** | **2.84 (2.60–3.11)** | **4.04 (3.65–4.46)** | **6.31 (5.60–7.11)** | **<0.0001** |
| **Model 2** | **1.34 (1.05–1.70)** | **1** | **1.15 (1.04–1.26)** | **1.32 (1.18–1.47)** | **1.57 (1.38–1.79)** | **2.09 (1.77–2.45)** | **<0.0001** |
| **Sex stratified sample** |  |  |  |  |  |  |  |
| **Male** |  |  |  |  |  |  |  |
| **cumulative mortality ,n(%)** | **78 (5.2)** | **603 (3.4)** | **1743 (5.2)** | **1502 (8.1)** | **834 (11.4)** | **420 (16.7)** |  |
| **Model 1** | **1.46 (1.16–1.85)** | **1** | **1.51 (1.38–1.66)** | **2.44 (2.22–2.68)** | **3.52 (3.17–3.91)** | **5.32 (4.69–6.02)** | **<0.0001** |
| **Model 2#** | **1.51 (1.17–1.94)** | **1** | **1.16 (1.05–1.29)** | **1.31 (1.17–1.47)** | **1.58 (1.38–1.81)** | **2.07 (1.75–2.45)** | **<0.0001** |
| **Female** |  |  |  |  |  |  |  |
| **cumulative mortality,n(%)** | **6 (0.8)** | **73 (1.0)** | **114 (1.6)** | **105 (3.3)** | **45 (3.9)** | **24 (7.2)** |  |
| **Model 1** | **0.42 (0.18–0.97)** | **1** | **1.65 (1.23–2.22)** | **3.56 (2.64–4.80)** | **3.95 (2.72–5.72)** | **8.01 (5.05–12.70)** | **<0.0001** |
| **Model 2#** | **0.50 (0.20–1.26)** | **1** | **1.01 (0.73–1.40)** | **1.43 (0.97–2.10)** | **1.48 (0.90–2.45)** | **2.39 (1.25–4.56)** | **0.034** |
| Model 1: unadjusted.  Model 2: adjusted for age, gender, diastolic blood pressure (DBP), triglycerides (TG), low-density lipoprotein cholesterol (LDL-C), high-density lipoprotein cholesterol (HDL-C), fasting blood glucose (FBG), body mass index (BMI), high-sensitivity C-reactive protein (hs-CRP), education level, physical activity, smoking status, alcohol consumption and use of antihypertensives.  Model 2#: adjusted for age, DBP, TG, LDL-C, HDL-C, FBG, BMI, hs-CRP, education level, physical activity, smoking status, alcohol consumption and use of antihypertensives. | | | | | | | |
