# Supplementary material for: Relationship between systolic blood pressure and all-cause mortality: a prospective study in a cohort of Chinese adults
Source: BMC Public Health. 2018 Jan 5;18:107. doi: 10.1186/s12889-017-4965-5 (PMC5756411; doi:10.1186/s12889-017-4965-5)
Supplement: Supplementary file 3 — Supplementary Table S3. Hazard ratios (HR) and 95% confidence intervals (95% CI) of all-cause mortality according to systolic blood pressure groups from 1 year after baseline. (DOC 52 kb) [file 12889_2017_4965_MOESM3_ESM.doc]

| **Supplementary Table S3 Hazard ratios (HR) and 95% confidence intervals (95% CI) of all-cause mortality according to systolic blood pressure groups from 1 year after baseline** | | | | | | | |
| --- | --- | --- | --- | --- | --- | --- | --- |
|  | **Systolic pressure groups** | | | | | | **P for trend** |
|  | **Q1** | **Q2** | **Q3** | **Q4** | **Q5** | **Q6** |  |
|  | **<100mm Hg** | **100–119mm Hg** | **120–139mm Hg** | **140–159mm Hg** | **160–179mm Hg** | **≥180mm Hg** |  |
| **Overall sample** |  |  |  |  |  |  |  |
| **cumulative mortality ,n(%)** | **80 (3.0)** | **691 (2.8)** | **1896 (4.6)** | **1665 (7.5)** | **917 (10.4)** | **458 (15.5)** |  |
| **Model 1** | **0.97 (0.77–1.22)** | **1** | **1.68 (1.54–1.83)** | **2.83 (2.59–3.09)** | **4.02 (3.64–4.43)** | **6.20 (5.51–6.97)** | **<0.0001** |
| **Model 2** | **1.24 (0.97–1.58)** | **1** | **1.15 (1.05–1.26)** | **1.33 (1.19–1.48)** | **1.60 (1.40–1.82)** | **2.11 (1.80–2.48)** | **<0.0001** |
| **Sex stratified sample** |  |  |  |  |  |  |  |
| **Male** |  |  |  |  |  |  |  |
| **cumulative mortality ,n(%)** | **74 (4.9)** | **613 (3.5)** | **1771 (5.2)** | **1547 (8.2)** | **860 (11.4)** | **431 (16.7)** |  |
| **Model 1** | **1.37 (1.08–1.74)** | **1** | **1.50 (1.37–1.65)** | **2.44 (2.22–2.68)** | **3.51 (3.17–3.90)** | **5.28 (4.67–5.97)** | **<0.0001** |
| **Model 2#** | **1.40 (1.09–1.80)** | **1** | **1.16 (1.06–1.29)** | **1.32 (1.18–1.48)** | **1.60 (1.40–1.82)** | **2.09 (1.78–2.47)** | **<0.0001** |
| **Female** |  |  |  |  |  |  |  |
| **cumulative mortality,n(%)** | **6 (0.8)** | **78 (1.0)** | **125 (1.8)** | **118 (3.5)** | **57 (4.5)** | **27 (7.2)** |  |
| **Model 1** | **0.40 (0.18–0.92)** | **1** | **1.67 (1.26–2.21)** | **3.56 (2.69–4.76)** | **4.35 (3.09–6.12)** | **7.66 (4.95–11.88)** | **<0.0001** |
| **Model 2#** | **0.47 (0.19–1.18)** | **1** | **1.02 (0.75–1.40)** | **1.45 (1.00–2.09)** | **1.64 (1.04–2.59)** | **2.42 (1.33–4.41)** | **0.010** |
| Model 1: unadjusted.  Model 2: adjusted for age, gender, diastolic blood pressure (DBP), triglycerides (TG), low-density lipoprotein cholesterol (LDL-C), high-density lipoprotein cholesterol (HDL-C), fasting blood glucose (FBG), body mass index (BMI), high-sensitivity C-reactive protein (hs-CRP), education level, physical activity, smoking status, alcohol consumption and use of antihypertensives.  Model 2#: adjusted for age, DBP, TG, LDL-C, HDL-C, FBG, BMI, hs-CRP, education level, physical activity, smoking status, alcohol consumption and use of antihypertensives. | | | | | | | |
