# Supplementary material for: Relationship between systolic blood pressure and all-cause mortality: a prospective study in a cohort of Chinese adults
Source: BMC Public Health. 2018 Jan 5;18:107. doi: 10.1186/s12889-017-4965-5 (PMC5756411; doi:10.1186/s12889-017-4965-5)
Supplement: Supplementary file 4 — Supplementary Table S4. Hazard ratios (HR) and 95% confidence intervals (95% CI) of all-cause mortality according to systolic blood pressure groups after exclusion of individuals who were older than 70 years. (DOC 52 kb) [file 12889_2017_4965_MOESM4_ESM.doc]

| **Supplementary Table S4 Hazard ratios (HR) and 95% confidence intervals (95% CI) of all-cause mortality according to systolic blood pressure groups after exclusion of individuals who were older than 70 years** | | | | | | | |
| --- | --- | --- | --- | --- | --- | --- | --- |
|  | **Systolic pressure groups** | | | | | | **P for trend** |
|  | **Q1** | **Q2** | **Q3** | **Q4** | **Q5** | **Q6** |  |
|  | **<100mm Hg** | **100–119mm Hg** | **120–139mm Hg** | **140–159mm Hg** | **160–179mm Hg** | **≥180mm Hg** |  |
| **Overall sample** |  |  |  |  |  |  |  |
| **cumulative mortality ,n(%)** | **60 (1.7)** | **527 (1.8)** | **1346 (3.0)** | **1004 (4.5)** | **564 (6.7)** | **306 (11.2)** |  |
| **Model 1** | **0.95 (0.73–1.24)** | **1** | **1.60 (1.45–1.77)** | **2.42 (2.18–2.69)** | **3.63 (3.22–4.09)** | **6.25 (5.42–7.19)** | **<0.0001** |
| **Model 2** | **1.27 (0.96–1.67)** | **1** | **1.20 (1.08–1.34)** | **1.39 (1.22–1.58)** | **1.84 (1.57–2.16)** | **2.86 (2.34–3.50)** | **<0.0001** |
| **Sex stratified sample** |  |  |  |  |  |  |  |
| **Male** |  |  |  |  |  |  |  |
| **cumulative mortality ,n(%)** | **54 (3.2)** | **455 (2.3)** | **1245 (3.3)** | **917 (4.8)** | **518 (7.3)** | **286 (12.0)** |  |
| **Model 1** | **1.35 (1.02–1.79)** | **1** | **1.46 (1.31–1.62)** | **2.11 (1.88–2.36)** | **3.20 (2.82–3.63)** | **5.41 (4.67–6.28)** | **<0.0001** |
| **Model 2#** | **1.47 (1.10–1.96)** | **1** | **1.23 (1.10–1.38)** | **1.40 (1.22–1.60)** | **1.86 (1.57–2.20)** | **2.88 (2.34–3.56)** | **<0.0001** |
| **Female** |  |  |  |  |  |  |  |
| **cumulative mortality,n(%)** | **6 (0.3)** | **72 (0.8)** | **101 (1.3)** | **87 (2.6)** | **46 (3.5)** | **20 (5.9)** |  |
| **Model 1** | **0.43 (0.19–0.99)** | **1** | **1.49 (1.10–2.02)** | **3.06 (2.24–4.18)** | **4.12 (2.85–5.97)** | **7.00 (4.26–11.48)** | **<0.0001** |
| **Model 2#** | **0.52 (0.21–1.30)** | **1** | **0.98 (0.70–1.36)** | **1.36 (0.90–2.05)** | **1.73 (1.04–2.89)** | **2.72 (1.37–5.42)** | **0.013** |
| Model 1: unadjusted.  Model 2: adjusted for age, gender, diastolic blood pressure (DBP), triglycerides (TG), low-density lipoprotein cholesterol (LDL-C), high-density lipoprotein cholesterol (HDL-C), fasting blood glucose (FBG), body mass index (BMI), high-sensitivity C-reactive protein (hs-CRP), education level, physical activity, smoking status, alcohol consumption and use of antihypertensives.  Model 2#: adjusted for age, DBP, TG, LDL-C, HDL-C, FBG, BMI, hs-CRP, education level, physical activity, smoking status, alcohol consumption and use of antihypertensives. | | | | | | | |
