# Supplementary material for: Relationship between systolic blood pressure and all-cause mortality: a prospective study in a cohort of Chinese adults
Source: BMC Public Health. 2018 Jan 5;18:107. doi: 10.1186/s12889-017-4965-5 (PMC5756411; doi:10.1186/s12889-017-4965-5)
Supplement: Supplementary file 5 — Supplementary Table S5. Hazard ratios (HR) and 95% confidence intervals (95% CI) of all-cause mortality according to systolic blood pressure groups stratified by age and smoking status. (DOC 67 kb) [file 12889_2017_4965_MOESM5_ESM.doc]

| **Supplementary Table S5 Hazard ratios (HR) and 95% confidence intervals (95% CI) of all-cause mortality according to systolic blood pressure groups stratified by age and smoking status** | | | | | | | |
| --- | --- | --- | --- | --- | --- | --- | --- |
|  | **Systolic pressure groups** | | | | | | **P for trend** |
|  | **Q1** | **Q2** | **Q3** | **Q4** | **Q5** | **Q6** |  |
|  | **<100mm Hg** | **100–119mm Hg** | **120–139mm Hg** | **140–159mm Hg** | **160–179mm Hg** | **≥180mm Hg** |  |
| **Overall sample** |  |  |  |  |  |  |  |
| **cumulative mortality ,n(%)** | **90 (3.3)** | **731 (2.9)** | **1972 (4.7)** | **1719 (7.7)** | **954 (10.7)** | **479 (16.0)** |  |
| **Model 1** | **1.03 (0.83–1.29)** | **1** | **1.65 (1.52–1.80)** | **2.76 (2.53–3.01)** | **3.95 (3.59–4.35)** | **6.12 (5.45–6.86)** | **<0.0001** |
| **Model 2** | **1.30 (1.03–1.64)** | **1** | **1.13 (1.03–1.24)** | **1.29 (1.17–1.44)** | **1.57 (1.39–1.78)** | **2.09 (1.79–2.44)** | **<0.0001** |
|  |  |  |  |  |  |  |  |
| **<60 ys** |  |  |  |  |  |  |  |
| **Male** | **26 (1.7)** | **317 (1.7)** | **813 (2.5)** | **517 (3.4)** | **272 (5.2)** | **152 (9.2)** |  |
| **Model 2 †** | **0.85 (0.52–1.38)** | **1** | **1.48 (1.25–1.74)** | **2.17 (1.77–2.65)** | **3.50 (2.72–4.51)** | **6.64 (4.81–9.16)** | **<0.0001** |
| **Female** | **6 (0.3)** | **60 (0.7)** | **61 (0.8)** | **53 (2.0)** | **27 (2.8)** | **12 (4.9)** |  |
| **Model 2 †** | **0.62 (0.24–1.58)** | **1** | **1.01 (0.66–1.54)** | **1.97 (1.16–3.33)** | **2.56 (1.28–5.14)** | **3.80 (1.46–9.90)** | **0.009** |
| **≥60ys** |  |  |  |  |  |  |  |
| **Male** | **57 (23.9)** | **334 (13.5)** | **1030 (14.2)** | **1079 (16.3)** | **623 (19.3)** | **300 (24.2)** |  |
| **Model 2 †** | **1.51 (1.12–2.05)** | **1** | **1.14 (0.99–1.30)** | **1.38 (1.20–1.59)** | **1.73 (1.47–2.03)** | **2.25 (1.85–2.75)** | **<0.0001** |
| **Female** | **1 (2.5)** | **20 (4.8)** | **68 (6.8)** | **70 (7.5)** | **32 (6.9)** | **15 (10.3)** |  |
| **Model 2 †** | **0.77 (0.18–2.32)** | **1** | **1.43 (0.86–2.39)** | **1.92 (1.11–3.33)** | **2.00 (1.05–3.82)** | **3.77 (1.70–8.36)** | **0.034** |
|  |  |  |  |  |  |  |  |
| **Smokers** |  |  |  |  |  |  |  |
| **Male** | **40 (3.8)** | **344 (3.0)** | **864 (4.4)** | **635 (6.5)** | **340 (9.6)** | **177 (14.1)** |  |
| **Model 2 ‡** | **1.14 (0.78–1.67)** | **1** | **1.14 (0.99–1.32)** | **1.11 (0.94–1.31)** | **1.38 (1.12–1.69)** | **1.72 (1.32–2.23)** | **<0.0001** |
| **Female** | **0 (0)** | **3 (1.7)** | **10 (6.3)** | **3 (3.4)** | **3 (8.8)** | **2 (12.5)** |  |
| **Model 2 ‡** | **-** | **-** | **-** | **-** | **-** | **-** | **-** |
| **Non-smokers** |  |  |  |  |  |  |  |
| **Male** | **43 (5.7)** | **307 (3.3)** | **979 (4.8)** | **961 (8.1)** | **555 (11.3)** | **275 (16.8)** |  |
| **Model 2 ‡** | **1.95 (1.38–2.76)** | **1** | **1.06 (0.91–1.22)** | **1.25 (1.07–1.46)** | **1.44 (1.20–1.73)** | **1.83 (1.47–2.29)** | **<0.0001** |
| **Female** | **7 (0.4)** | **77 (0.9)** | **119 (1.5)** | **120 (3.4)** | **56 (4.0)** | **25 (6.6)** |  |
| **Model 2 ‡** | **0.78 (0.35–1.72)** | **1** | **0.91 (0.66–1.27)** | **1.30 (0.89–1.90)** | **1.34 (0.84–2.13)** | **1.94 (1.04–3.61)** | **0.078** |
| Model 1: unadjusted.  Model 2: adjusted for age, gender, diastolic blood pressure (DBP), triglycerides (TG), low-density lipoprotein cholesterol (LDL-C), high-density lipoprotein cholesterol (HDL-C), fasting blood glucose (FBG), body mass index (BMI), high-sensitivity C-reactive protein (hs-CRP), education level, physical activity, smoking status, alcohol consumption and use of antihypertensives.  Model 2 †: adjusted for DBP, TG, LDL-C, HDL-C, FBG, BMI, hs-CRP, education level, physical activity, smoking status, alcohol consumption and use of antihypertensives.  Model 2 **‡**: adjusted for age, DBP, TG, LDL-C, HDL-C, FBG, BMI, hs-CRP, education level, physical activity, alcohol consumption and use of antihypertensives. | | | | | | | |
